# Supplementary material for: Guanine Radicals Generated in Telomeric G-Quadruplexes by Direct Absorption of Low-Energy UV Photons: Effect of Potassium Ions
Source: Molecules. 2020 Apr 30;25(9):2094. doi: 10.3390/molecules25092094 (PMC7248781; doi:10.3390/molecules25092094)
Supplement: Supplementary file 1 [file molecules-25-02094-s001.pdf]

# Guanine Radicals Generated in Telomeric G-quadruplexes by Direct Absorption of Low-Energy UV Photons: Effect of Potassium Ions

**Evangelos Balanikas [1], Akos Banyasz[1,2], Gérard Baldacchino[1], and Dimitra Markovitsi[1,*]**

[1] LIDYL, CEA, CNRS, Université Paris-Saclay, F-91191 Gif-sur-Yvette, France; vangelis.balanikas@cea.fr (E.V.); akos.banyasz@ens-lyon.fr (A.B.); gerard.baldacchino@cea.fr (G.B.)

[2] Univ Lyon, ENS de Lyon, CNRS UMR 5182, Université Claude Bernard Lyon 1, Laboratoire de Chimie, F-69342 Lyon, France (A.B.)

* Correspondance: dimitra.markovitsi@cea.fr (D.M.). TEL : +33169084644

Academic Editors: Virginie Lyria-Lhiaubet, Iñaki Tuñon, Daniel Roca-Sanjuan

# Supplementary Information

---

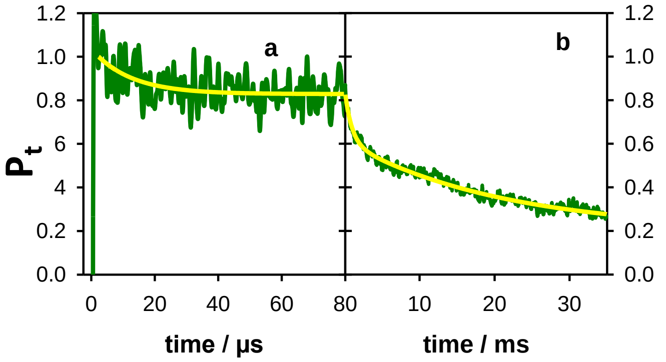


**Figure S1.** Survival probability $P_t$ of the total **G** radical population in **TEL21/Na⁺**, estimated by the transient absorption signals at 512 nm. Yellow lines correspond to fits with bi-exponential model functions.

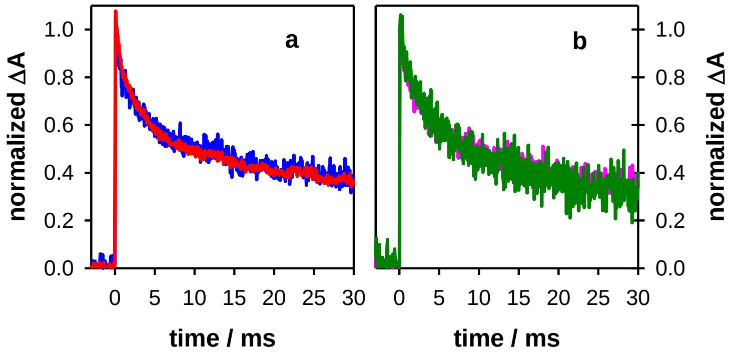


**Figure S2**. Transient absorption signals recorded for **TEL21/K**$^{+}$ at 365 nm (**a**) and 395 nm (**b**). (**a**) Aerated solutions; incident laser pulses: 3 mJ (blue) and 6 mJ (red). (**b**) Incident laser pulses: 6 mJ; pink: argon-saturated solutions (pink) and aerated (green) solutions.

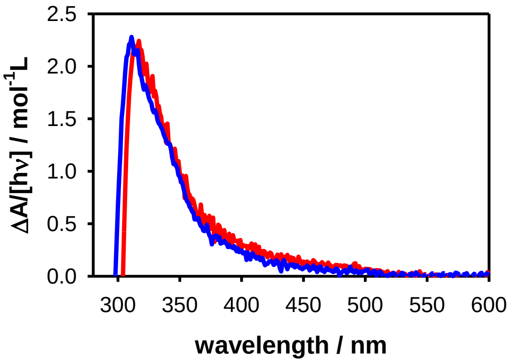


**Figure S3**. Comparison of the steady-state differential absorption spectra obtained for **TEL21/K**$^{+}$ (blue) and **TEL21/Na**$^{+}$ (red; intensity divided by 2.3); [hν] is the total concentration of photons absorbed by the solution.

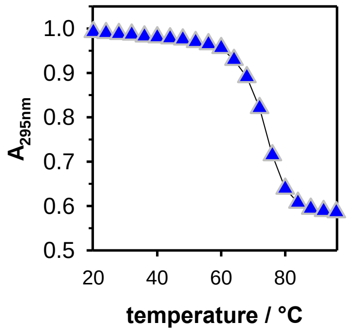


**Figure S4.** Absorbance determined at 295°C for **TEL21/K**$^{+}$ as a function of temperature.
